# Elevated cellular PpIX potentiates sonodynamic therapy in a mouse glioma stem cell-bearing glioma model by downregulating the Akt/NF-κB/MDR1 pathway

**DOI:** 10.1038/s41598-021-93896-0

**Published:** 2021-07-23

**Authors:** Kenji Shono, Yoshifumi Mizobuchi, Izumi Yamaguchi, Kohei Nakajima, Yuri Fujiwara, Toshitaka Fujihara, Keiko Kitazato, Kazuhito Matsuzaki, Yoshihiro Uto, Oltea Sampetrean, Hideyuki Saya, Yasushi Takagi

**Affiliations:** 1 Department of Neurosurgery, Tokushima University Graduate School of Biomedical Sciences, 3-18-15 Kuramoto-cho, Tokushima, 770-8503 Japan; 2 Graduate School of Technology, Industrial and Social Science, Tokushima University, Tokushima, Japan; 3 Division of Gene Regulation, Institute for Advanced Medical Research, Keio University School of Medicine, Tokyo, Japan

**Keywords:** Cancer, CNS cancer

## Abstract

Glioblastoma (GBM) has high mortality rates because of extreme therapeutic resistance. During surgical resection for GBM, 5-aminolevulinic acid (5-ALA)-induced protoporphyrin IX (PpIX) fluorescence is conventionally applied to distinguish GBM. However, surgical intervention is insufficient for high invasive GBM. Sonodynamic therapy (SDT) combined with low-intensity ultrasonication (US) and PpIX, as a sonosensitizer, is an emerging and promising approach, although its efficacy is limited. Based on our previous study that down-regulation of multidrug resistant protein (MDR1) in GBM augmented the anti-tumor effects of chemotherapy, we hypothesized that elevation of cellular PpIX levels by down-regulation of MDR1 enhances anti-tumor effects by SDT. In high invasive progeny cells from mouse glioma stem cells (GSCs) and a GSC-bearing mouse glioma model, we assessed the anti-tumor effects of SDT with a COX-2 inhibitor, celecoxib. Down-regulation of MDR1 by celecoxib increased cellular PpIX levels, as well as valspodar, an MDR1 inhibitor, and augmented anti-tumor effects of SDT. MDR1 down-regulation via the Akt/NF-κB pathway by celecoxib was confirmed, using an NF-κB inhibitor, CAPÉ. Thus, elevation of cellar PpIX by down-regulation of MDR1 via the Akt/NF-κB pathway may be crucial to potentiate the efficacy of SDT in a site-directed manner and provide a promising new therapeutic strategy for GBM.

## Introduction

Glioblastoma (GBM) is the most common primary malignant brain tumor and has high recurrence and mortality rates^1,2^. Various current therapeutic approaches, including surgery, chemotherapy, radiotherapy, and immunotherapy, are clinically applied for treating GBMs^3,4^. However, surgical intervention is insufficient for eradicating GBM because of its extremely high invasiveness^3^. Chemotherapy and radiotherapy not only damage tumor cells but also partly harm normal cells. Furthermore, tumor cells acquire chemo-resistance during treatment, thus deterring GBM treatment. Though various approaches including neoadjuvant anti-PD-1 immunotherapy have been studied^5,6^, novel therapeutic strategies are required to improve the prognosis of GBM patients.

Sonodynamic therapy (SDT) is a promising and emerging noninvasive approach for cancer treatment. SDT involves a combination of ultrasonication (US) via low-intensity ultrasound imaging and specialized chemical agents known as sonosensitizers including 5-aminolevulinic acid (5-ALA)-induced protoporphyrin IX (PpIX)^7,8^. After oral administration, 5-ALA can accumulate in GBM and surrounding infiltrating cancer cells such as glioma stem cells (GSCs) outside of the tumor bulk. Once formed, PpIX emits fluorescence at a peak wavelength of 635 nm after excitation with light near the Soret band peak around 410 nm^9^. Microbubbles generated during US implode and release markedly high energy and initiate the emission of sonoluminescence light, subsequently leading to the generation of reactive oxygen species (ROS). The anti-tumor effect of SDT is primarily attributed to the generated ROS, suggesting that mitochondrial ROS generation is promoted under the presence of PpIX upon SDT^10^, resulting in severe damage to tumor cells through hydrodynamic shear forces and selectively destroying tumor cells^10,11^. Currently, 5-ALA is commonly administered to visualize GBM during resection surgery. It is selectively taken up by tumor cells upon delivery and localizes to the mitochondria, being converted to PpIX^10^. Because of its specificity and effectiveness towards highly invasive GBM cells^11^, SDT might potentially be a novel strategy for glioma therapy. However, SDT monotherapy cannot completely eradicate tumors. Since the efficacy of SDT depends on cellular PpIX levels to induce intrinsic caspase-dependent apoptosis^9–13^, it may be necessary to elevate the cellular PpIX level to promote the effects of SDT with new methods, including high-intensity focused US^14^.

P-Glycoprotein, referred to as multidrug resistance receptor (MDR1), is a transmembrane glycoprotein functioning as an efflux pump and conferring multidrug resistance in brain tumors^15,16^. We have previously reported high expression of MDR1 in GBM and demonstrated that down-regulation of MDR1 via Akt/NF-κB pathways upon transfection of the Ad-DKK3 gene augmented the anti-tumor effects of temozolomide in GBM cells and in a GBM-xenograft model^17^. We also reported that a selective cyclooxygenase-2 (COX-2) inhibitor, celecoxib exerted anti-tumor effects associated with the down-regulation of Akt/NF-κB pathways in mouse glioma stem cells (GSCs) and GSCs-bearing glioma model^18,19^. Based on these findings, we hypothesized that down-regulation of MDR1 by celecoxib via Akt/NF-κB pathways may promote the uptake of 5-ALA into GBM, thereby elevating cellular PpIX levels and enhance the anti-tumor effects of SDT.

This study shows that elevation of cellular PpIX through celecoxib-mediated MDR1 down-regulation potentiates anti-tumor effects of SDT in a mouse GSC-bearing malignant glioma model which is highly invasive and similar to GBM in many respects^20,21^.

## Results

### Effects of SDT on GSC cell lines

**Figure 1:**
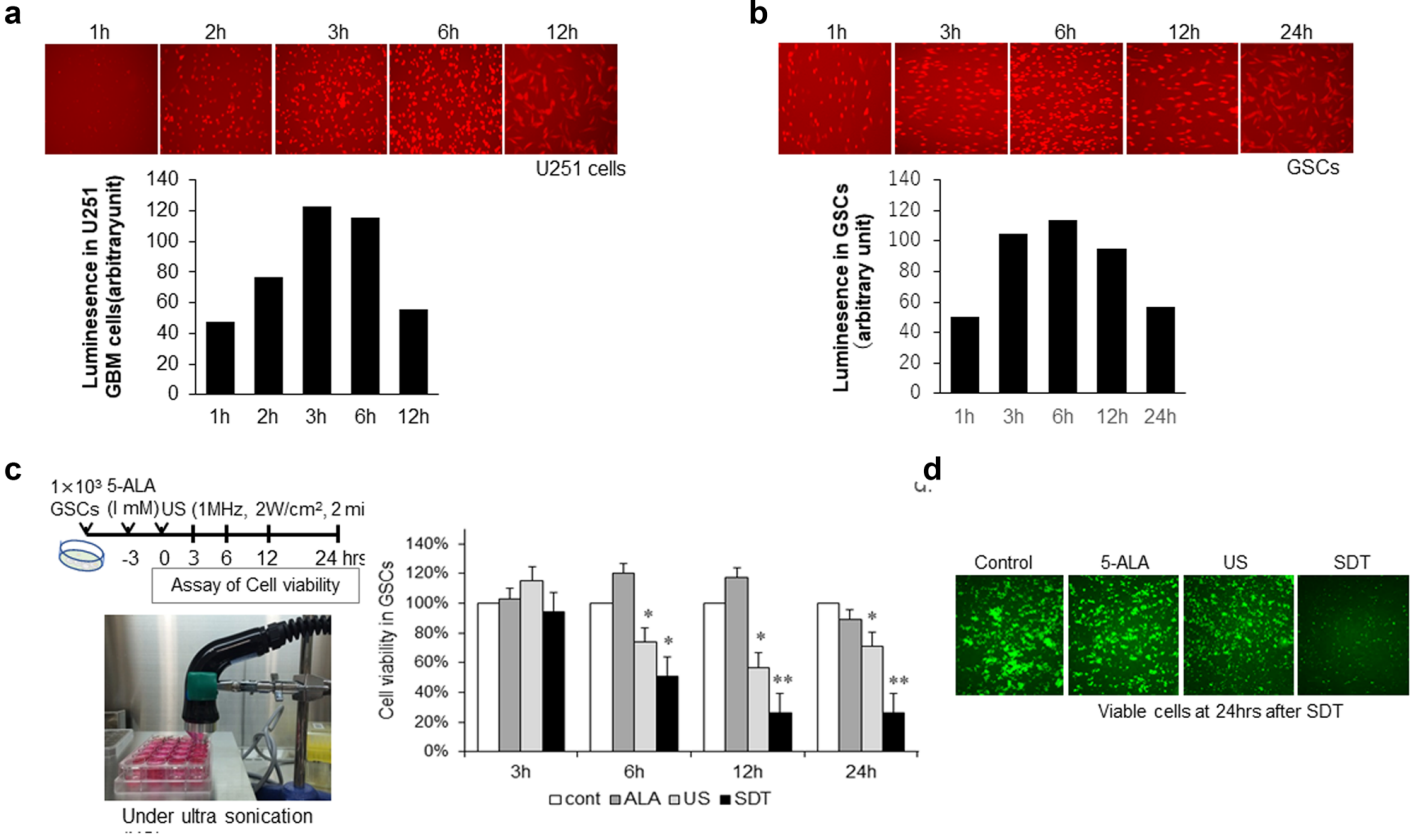
Cell viability by sonodynamic therapy (SDT) in mouse glioma stem cells (GSCs). (**a**) Changes of luminescence images and intensity in human glioblastoma U251 cells for 12 h after treatment with 1 μM 5-aminolevulinic acid (5-ALA) as a sonosensitizer. (**b**) Changes of luminescence images and intensity in mouse GSCs for 24 h after treatment with 5-ALA. (**c**) Protocol and photos under ultrasonication (US) and changes in cell viability. Time course of cell viability by 5-ALA, US, SDT; a combination of low-intensity US (1 MHz, 2 W/cm^2^, 2 min) after treatment with 1 μM 5-ALA. Each column data indicates mean ± SD (n = 6). *p < 0.05, **p < 0.01 by Turkey–Kramer versus non-treated control. (**d**) Representative images of viable GFP-labeled GSCs in control and treatment with 5-ALA or US alone and SDT.

Based on clinical usage in GBM patients, we first confirmed the time course of PpIX fluorescence in a GBM cell line, U251 (Fig. 1a).and in GSCs (Fig. 1b). Since the luminescence intensity peaked at 3 h after treatment with 5-ALA and was retained for 12 h in both cell lines (Fig. 1a,b), we decided to perform US (1 MHz, 2 W/cm^2^ for 2 min) at 3 h after treatment with 1 μM 5-ALA, based on previous study^10,12^. The effects of SDT in GSCs was assessed at 3, 6, 12, 24 and 48 h after SDT and compared with non-treatment control, treatment with 5-ALA or US alone (Fig. 1c,d). A significant reduction of cell viability was observed at 6–48 h after SDT.

### Anti-tumor effects of SDT in the mouse GSC-bearing glioma model

**Figure 2:**
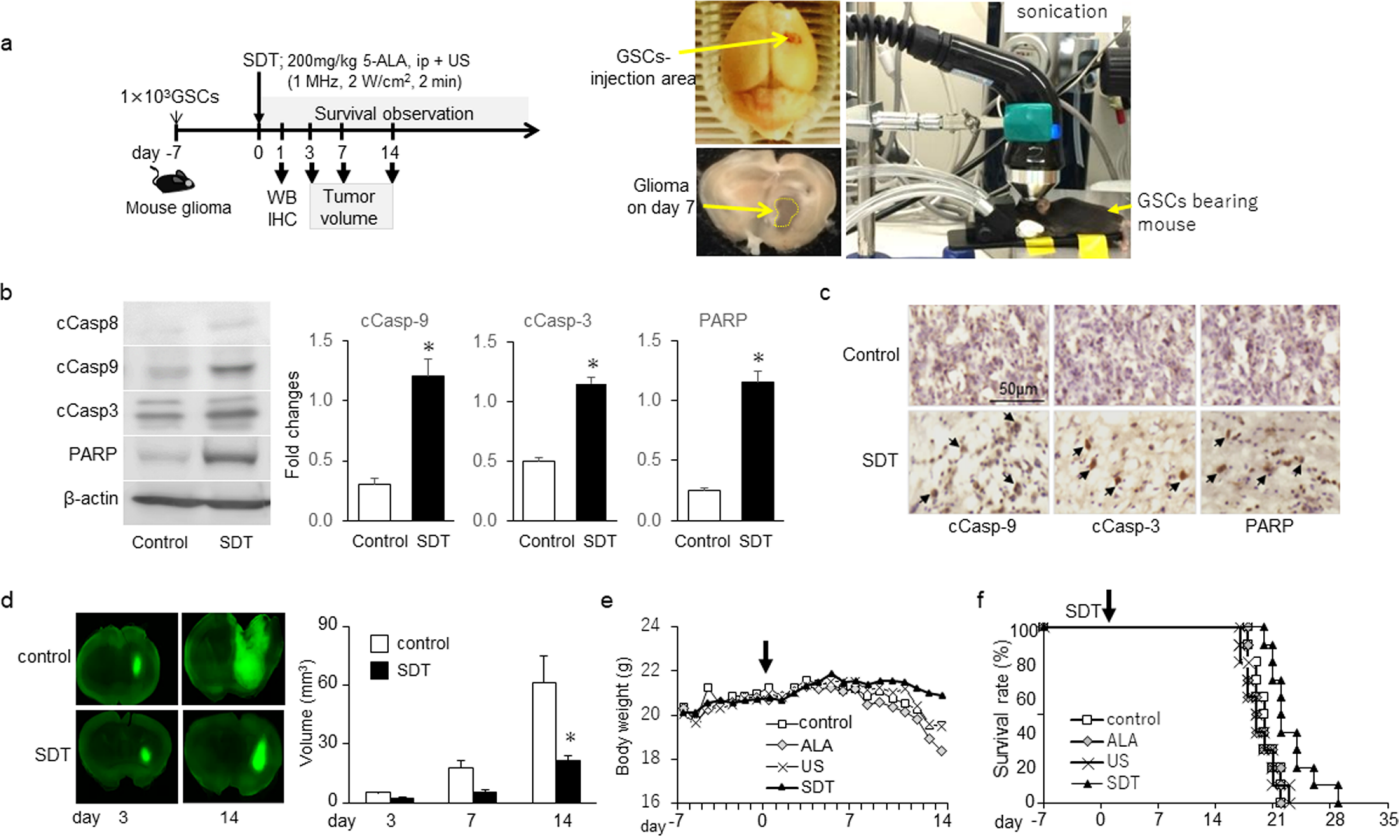
Anti-tumor effects of SDT in the mouse GSC-bearing glioma model. (**a**) Protocol in mouse GSC-bearing glioma model; SDT performed 7 days after injection of GSCs. The mice subjected to US 1 MHz, 2 W/cm^2^ 2 min) at 3 h after injection of 200 mg/kg 5-ALA. Photographs indicate mice subjected to SDT, GSC injection area, and glioma. (**b**) Representative western blot analysis on day 1 after SDT compared to non-treatment control in a GSC-bearing mouse glioma model. Each column indicates mean ± SD (n = 4). *p < 0.05 by Student’s t-test. (**c**) DAB immunohistochemistry of apoptosis-related molecules on day 1 after SDT in a glioma-bearing mouse. Each specimen was co-stained with hematoxylin. Arrows point to positive cells. (**d**) GFP-labeled GSCs and tumor volume in brain on days 3–14 after SDT. Each column data indicates mean ± SD (n = 6). *p < 0.05 by Student’s t-test. (**e**) Changes in body weight before and after SDT. (**f**) Kaplan–Meier survival estimate (%), no significant difference by log-rank test (n = 10).

We further examined the effects of SDT in the mouse GSC-bearing glioma model (Fig. 2a). SDT was performed 7 d after GSC injection. One day after SDT, we observed up-regulation of cCasp-9, -3 and PARP but not cCasp-8 by western blot analysis (Fig. 2b) and immunohistochemistry (Fig. 2c), compared to non-treatment control, indicating the induction of intrinsic apoptosis by SDT. SDT significantly decreased the tumor size (Fig. 2d) without affecting body weights 14 d after GSC injection (Fig. 2e) but did not significantly extend the survival period compared with the values in other groups (Fig. 2f). The anti-tumor effects of single SDT may be limited in this model.

### An increase in cellular PpIX via celecoxib-mediated MDR1 down-regulation augmented the anti-tumor effect of SDT

**Figure 3:**
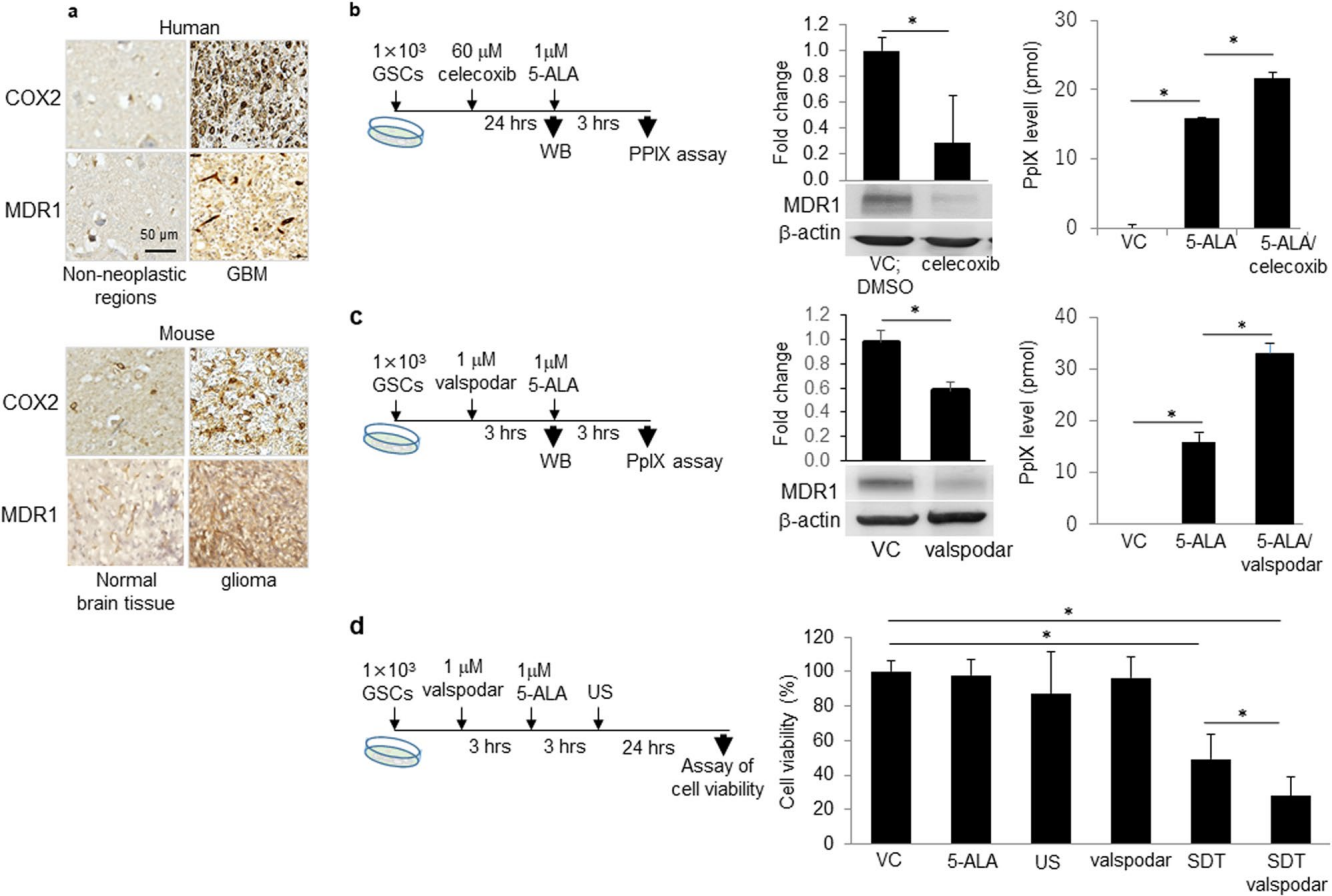
Elevation of PpXI through down-regulation of MDR1 leading to low cell viability in GSCs. (**a**) Representative expression of COX-2 and MDR1 by DAB stain in human GBM and GSCs-bearing mouse glioma model and each normal tissue. (**b**) Changes in MDR1 expression and PpIX level treated with celecoxib and 5-ALA. Western blot analysis performed at 24 h after treatment with 60 μM celecoxib and compared with DMSO as a vehicle control (VC). PpXI levels were determined at 3 h after 1 μM 5-ALA. Each column indicates mean ± SD values (n = 4). (**c**) Changes in MDR expression and PpIX levels upon treatment with valspodar as a MDR1 inhibitor and 5-ALA. Western blot analysis performed at 3 h after valspodar and PpXI level determined at 3 h after 5-ALA. (**d**) Cell viability of GSCs treated with 1 μM 5-ALA at 3 h after treatment with 1 μM valspodar. US was performed at 3 h after 5-ALA. Each column indicates mean ± SD values (n = 8). *p < 0.05 by Student’s t-test versus VC (2 groups) or Turkey–Kramer’s test versus others (> 3 groups).

To enhance the anti-tumor effects of SDT, we examined whether MDR1 down-regulation can increase the cellular PpIX levels after 5-ALA injection. Based on our previous findings in human glioma cells^17^, we first observed that the expression levels of COX-2 and MDR1 in human glioblastoma and the mouse GSC-bearing glioma model were higher than those in normal brain tissue (Fig. 3a). Next, using a COX-2 inhibitor, celecoxib, we assessed the effects on the MDR1 expression in GSCs.

Treatment with celecoxib 60 μM (IC_50_ dose) decreased MDR1 expression levels compared to diluted DMSO as vehicle control and the PpIX levels were significantly higher upon combination treatment with 1 μM 5-ALA and celecoxib than with 5-ALA monotherapy (Fig. 3b). To confirm the effects of MDR1 down-regulation on PpIX levels, we examined the effects of an MDR1 inhibitor, valspodar (Fig. 3c). MDR1 was down-regulated by 1 μM valspodar (Fig. 3c) and cellular PpIX levels increased as expected, resulting in the enhanced anti-tumor effects of SDT by the combination of valspodar and SDT in GSCs (Fig. 3d). The increase in cellular PpIX levels through MDR1 decreased by celecoxib may be at least partly attributable to the enhancement of the anti-tumor effects of SDT.

### Combination therapy with SDT and celecoxib enhanced apoptosis induction, thereby enhancing anti-tumor effects in the mouse GSC-bearing glioma model

**Figure 4:**
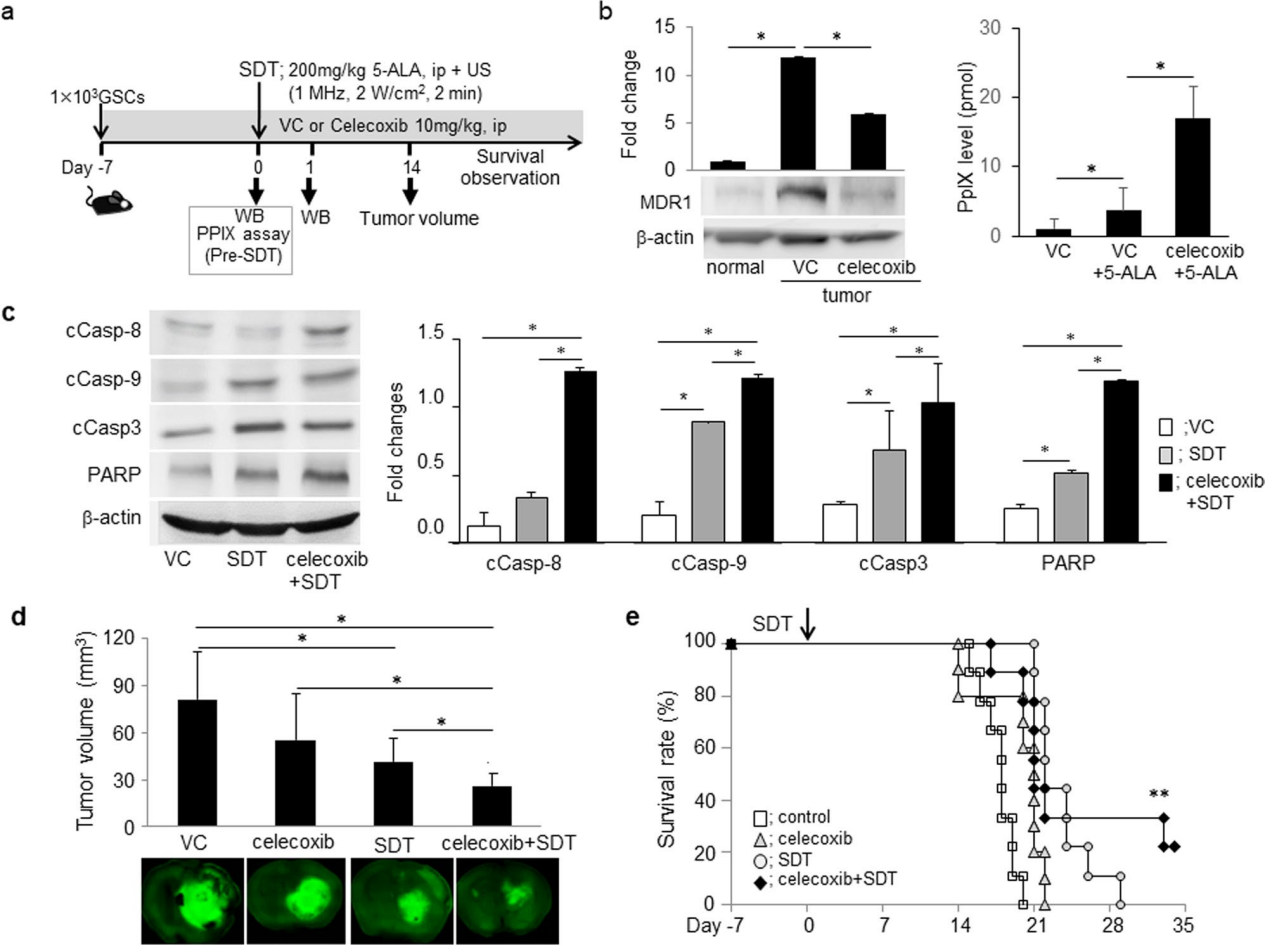
Elevation of cellular PpXI levels via MDR1 down-regulation by celecoxib augmented anti-tumor effects by SDT in the mouse glioma model. (**a**) Protocol: celecoxib was continuously injected during the survival period after GSC injection. (**b**) Expression of MDR1 by western blot analysis and PpIX level 3 h after SDT at 7 days after GSCs injection. The MDR1 expression was compared with normal- and tumor brain tissue treated with celecoxib or a vehicle control (VC) included DMSO/HBC. Each data indicates mean ± SD (n = 4). *p < 0.05 by Turkey–Kramer’s test versus others (> n = 3). (**c**) Expression analysis of apoptosis-related molecules by western blotting at day 1 after SDT (each, n = 4). (**d**) Tumor volume 14 days after SDT. (each, n = 6). (**e**) Kaplan–Meier survival estimate (%) and survival period prolonged by the combination therapy with SDT and celecoxib. **p < 0.01 versus other groups by the log-rank test (n = 9–10).

To further confirm whether SDT with down-regulation of MDR1 by celecoxib augments anti-tumor effects, we assessed the efficacy in the mouse GSC-bearing glioma model (Fig. 4a). Compared to normal tissue, the expression of MDR1 was elevated in brain tumor tissue after GSCs injection, which was reduced upon treatment with 10 mg/kg celecoxib for 7 days before SDT (Fig. 4b). Concurrent with this finding, cellular PpIX levels in brain tumor after 5-ALA injection were significantly increased (Fig. 4b). The elevation of cellular PpIX levels upon combination therapy with SDT and celecoxib escalated the induction of intrinsic and extrinsic apoptosis 1 day after SDT (Fig. 4c), thus decreasing the tumor volume (Fig. 4d) and prolonging survival (Fig. 4e). The combination therapy with SDT and celecoxib may be a promising means for GBM treatment.

### Enhancement of anti-tumor effects by SDT was attributed to celecoxib-mediated MDR1 down-regulation through the AKT/NF-κB pathway

**Figure 5:**
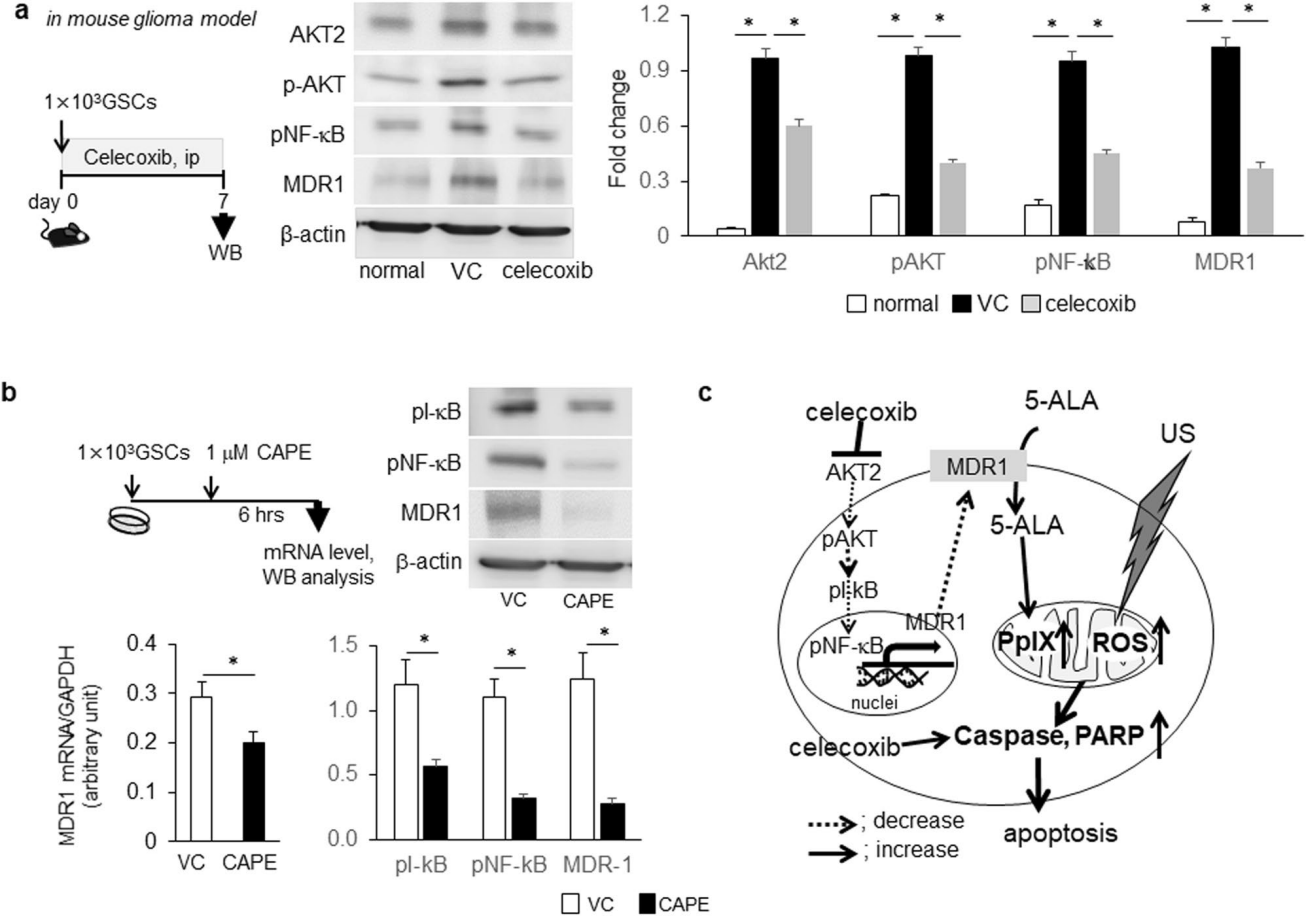
Anti-tumor effects of SDT combined with the inhibition of the AKT/NF-κB/MDR1 pathway by celecoxib in the GSCs-bearing mouse glioma model. (**a**) AKT/NF-κB signaling pathways and MDR1 expression upon western blotting after consecutive treatment with celecoxib for 7 days in the GSC-bearing mouse glioma model. Each expression level was compared between normal brain and tumor brain tissue treated with vehicle control (VC) or celecoxib. Each data indicates mean ± SD (n = 6). (**b**) Protein and mRNA levels of MDR1 in GSCs treated with or without 1 μM CAPE, a potent and a specific inhibitor of NF-κB activation. (each, n = 6). (**c**) Schematic representation of the anti-tumor effects upon combination therapy with SDT and celecoxib.

To clarify the mechanisms underlying celecoxib-mediated MDR1 down-regulation, we treated (i.p.) the mouse GSC-bearing glioma model with 10 mg/kg celecoxib for 7 days (Fig. 5a). AKT2, pAkt, pNF-κB, and MDR1 were significantly up-regulated in the glioma model than in normal tissue and were attenuated through celecoxib treatment (Fig. 5a). Furthermore, to confirm the transcriptional regulation of MDR1, we used a proteasome inhibitor, caffeic acid phenethyl ester (CAPE), which inhibits the phosphorylation of inhibitor of κB (I-κB), thus facilitating the phosphorylation of NF-κB (pNF-κB) and regulating the transcription of target genes. In GSCs, up-regulation of MDR1 mRNA and protein was significantly attenuated upon treatment with 1 μM CAPE, accompanied by the reduction of pI-κB and pNF-κB (Fig. 5b), suggesting transcriptional regulation of MDR1 through NF-κB. Together, celecoxib-mediated MDR1 down-regulation through the Akt/NF-κB pathway may increase cellular PpIX levels by increasing the intratumoral uptake of 5-ALA, thus promoting apoptosis induction via SDT (Fig. 5c). MDR1 down-regulation may be attributable to the improvement of not only chemo-resistance, but also the therapeutic efficacy of SDT in GBM.

## Discussion

In this study, we initially show that combination of SDT and celecoxib to down-regulate MDR1 augmented the anti-tumor effects in GSCs and the mouse GSC-bearing malignant glioma model indicating invasive and therapeutic resistance. First, we confirmed that SDT monotherapy exerted anti-tumor effects in GSCs and GSCs-bearing glioma model; however, it displayed transient and limited efficacy. Since we have demonstrated the anti-tumor effects by a COX2 inhibitor, celecoxib^18,19^, we assessed the efficacy of SDT combined with pre-treatment by celecoxib. Expectedly, the pre-treatment by celecoxib decreased the expression of MDR-1 and elevated the cellular PpIX levels induced by 5-ALA, resulting in the enhanced anti-tumor effects of the combination therapy in the GSC-bearing glioma model. Next, to confirm the mechanisms underlying the downregulation of MDR-1 by celecoxib, we used valspodar as an MDR-1 inhibitor. It decreased the expression of MDR-1 and elevated the cellular PpIX levels induced by 5-ALA, thereby reducing cell viability. Finally, we verified the down-regulation of MDR-1 by celecoxib via Akt/NF-κB pathway, using CAPE, a NF-κB inhibitor. The combination therapy of SDT and celecoxib enhanced the induction of both intrinsic and extrinsic apoptosis pathways in the GSCs-bearing malignant glioma model. Taken together, combination therapy with SDT and celecoxib may be a promising strategy to retard GBM recurrence.

SDT has been developed as a novel promising noninvasive approach derived from photodynamic therapy (PDT)^22,23^. Because PDT is tightly focused with a short penetration depth of light in soft tissue up to several tens of centimeters, PDT is not effective for the treatment of deep-seated tumors^24,25^. In contrast, SDT is anticipated to overcome the major limitation of PDT. It may be a promising treatment method for brain tumors and other tumors^26^. PpIX, converted from 5-ALA in mitochondria, is used in heme formation in a reaction catalyzed by the enzyme ferrochelatase. In GBM, the decrease of ferrochelatase may contribute to the accumulation of PpIX within gliomas and GSCs and lead to its preferential localization in tumor^9^. Although we did not assess the effects of ferrochelatase, we suggested that the increased cellular PpIX level may be at least partly attributable to the uptake of 5-ALA into brain or GSCs through down-regulation of MDR-1 by celecoxib, thereby enhancing anti-tumor effects. Because GBM occurs in the brain parenchyma surrounded by the skull and differs from other epithelial cancers, thus far, SDT has been performed for GBM patients only during surgical intervention^27^. Although the ultrasound instrument allows the repeated application of STD, this technique is limited to only a few institutes. Therefore, to enhance the repeatability of SDT, the development of feasible and safe equipment with new sonosensitizers^28^ are urgently required.

MDR1 (ABCB1) gene encodes P-glycoprotein, a drug transporter that is a critical component of the blood-brain barrier, which prevents entry of many potentially toxic compounds into the central nervous system^29^. We previously reported that the reduction of MDR1 upon transfection of DKK3 gene in human glioma cells enhanced the anti-tumor effects of temozolomide^17^. Concurrent with a previous report indicating increased mRNA levels of MDR1 in COX-2-overexpressing cells, the present study showed that MDR1 was highly up-regulated and COX-2 was overexpressed in GSC-derived glioma tissue and in human glioma tissue. COX-2, an inducible form of the enzyme catalyzing the first step in prostanoid synthesis, has been reported to be overexpressed in various tumors and possesses proangiogenic and anti-apoptotic properties. MDR1 is expressed in normal liver and kidney tissue, where it functions to actively transport lipophilic xenobiotic compounds and serves as an efflux pump for chemotherapeutic agents^30^. Thus, up-regulation of MDR1 and COX-2 may be associated with not only chemotherapeutic resistance but also with the limited uptake of 5-ALA in GSCs and the mouse GSC-bearing glioma model. To confirm the enhanced efficacy of SDT through the cellular up-take of 5-ALA upon MDR1 down-regulation in GSCs, we treated GSCs with a COX-2 inhibitor, celecoxib, or an MDR1 inhibitor, valspodar. As expected, cellular PpIX levels increased upon MDR1 down-regulation by each chemical compound in GSCs. Whereas celecoxib is applicable in a clinical setting, valspodar has not shown evidence of clinical efficacy. Therefore, we used celecoxib but not valspodar to reduce MDR1 in our *in-vivo* study and confirmed the efficacy of SDT enhanced by celecoxib.

Although glioma-associated macrophages may take up large amounts of 5-ALA, we did not directly assess the effects of celecoxib on macrophages. However, we previously verified the reduction on chemokines/its receptors (CCL2/CCR2 and CXCL10/CXCR3) by celecoxib^18^. The reduction of these molecules may inhibit the recruitment of macrophages into the tumor, resulting in anti-tumor effects in the GSC-bearing glioma model. Therefore, the ability to take up 5-ALA into macrophages may be limited in the presence of celecoxib. Taken together, treatment with celecoxib before SDT may contribute to increase the uptake of 5-ALA into the tumor, thereby enhancing the anti-tumor effects.

In addition, MDR1 expression is associated with several cellular signaling pathways and protein kinases, chaperons, ubiquitin-related enzymes, and transcription factors^31^. As the COX-2 inhibitor induced apoptosis by inhibiting the AKT pathway in low-grade glioma cells in a previous study^18^, we observed MDR1 down-regulation through the Akt/NF-κB pathway upon celecoxib treatment. Furthermore, we observed the down-regulation of MDR1 by a NF-κB inhibitor, CAPE. Thus, celecoxib may affect both apoptosis induction and MDR1 down-regulation by inhibiting the Akt/NF-κB pathway, resulting in an enhanced anti-tumor effects of not only SDT but also other anti-tumor agents.

This study has some limitations. Within a short period after SDT, apoptosis induction was enhanced in combination with celecoxib, and the cell viability was decreased. Therefore, we need the assessment of anti-tumor effects through repeated SDT upon combination during a longer period treatment with celecoxib. We previously confirmed that on combination treatment with 5-ALA and US, ROS generation and anti-tumor effects were greater than those upon monotherapy^10^. Unfortunately, in the current study we did not assess ROS generation, although the elevated cellular PpIX level was confirmed. Further investigation is necessary to determine whether elevated PpIX in combination therapy with SDT and celecoxib is associated with the enhanced ROS generation, thereby leading to the augment of the anti-tumor effects. SDT activates the mitochondrial caspase pathway and down-regulates ATP-binding cassette transporters such as MDR1, thus selectively improving the uptake of chemotherapeutic drugs into tumor cells and reducing the toxic effects on normal cells and tissues^31,32^. Therefore, sequential treatment with celecoxib after SDT may improve the uptake of celecoxib by itself into glioma cells, contributing to the prolonged survival in our glioma model. Although combination therapy with SDT and celecoxib may exert various synergistic therapeutic effects on GBM, we cannot exclude the favorable effects of celecoxib after SDT beyond MDR1 down-regulation^33^. Further studies are required to develop clinically applicable, handy, repeatable, and feasible devices for SDT.

In conclusion, the combination therapy with SDT and celecoxib resulted in enhanced anti-tumor efficacy among GSCs and a mouse GSC-bearing glioma model. MDR1 down-regulation via the Akt/NF-κB pathway may be a promising mean for treatment of GBM patients. Our results warrant for further verification of the anti-tumor effects of combination therapy with SDT and other chemotherapeutic agents and the development of new US systems capable of penetrating the skull.

## Method

### Study approval and informed consent

This study was approved by the ethical review board of Tokushima University Hospital for human study and the ethics committee of Tokushima University Graduate School of Biomedical Sciences, Tokushima, Japan. Human tissue samples were obtained during routine clinical procedures after informed consent including the use of tissue sample from patients with brain tumors at the Department of Neurosurgery, Tokushima University Hospital. For the use of samples obtained from patients, we have obtained a statement attesting to informed consent for all patients with or without neurosurgery in our department. Each sample was fixed in 4% formalin in phosphate-buffered saline (PBS) and processed for paraffin embedding. The samples were classified by neuropathologists in accordance with the WHO classification of brain tumors. Sections from non-neoplastic regions (NNRs) were purchased from BioChain Institute (Newark, NJ, USA). The study was performed in accordance with the tenets of the Declaration of Helsinki. All animal experiments were approved and performed in accordance with the animal care guidelines of Tokushima University.

### Cell lines

Human GBM cell line U251MG were purchased from American Type Culture Collection (Manassas, VA, USA) and cultured in RPMI-1640 medium (Invitrogen, NJ, USA) with 10% fetal bovine serum (GIBCO-BRL, NY, USA) at 37 °C in an atmosphere of 5% CO_2_ and 95% humidified air. Mice GSCs were established and provided by OS and HS, Keio University^19,20^. GSCs were cultured in Dulbecco’s Modified Eagle’s medium/nutrient mixture F-12 Ham (Sigma-Aldrich, St. Louis, MO, USA) supplemented with 20 ng/ml recombinant human epidermal growth factor (PeproTech, Rocky Hill, NJ, USA), 20 ng/ml recombinant human basic fibroblast growth factor (PeproTech), B-27 supplement without vitamin A (Life Technologies, Carlsbad, CA, USA), 200 ng/ml heparin sulfate, 100 U/ml penicillin, and 100 μg/ml streptomycin (Nacalai Tesque, Kyoto, Japan).

### Cell viability assay

GSCs (1 × 10^3^ cells/well) were plated in 96-well tissue culture plates. To enumerate viable cells, the conversion of WST-8 to formazan by metabolically active cells was quantified using WST-8 reagent (Dojindo, Osaka, Japan) on a microplate reader (Infinit F200 PRO, TECAN) at 450 nm. We used PBS-treated cells as the control to represent 100% viability and the percent viability was determined in each treatment.

### Establishment of the animal model and assessment of anti-tumor effects

All experimental protocol was approved by Tokushima University institutional committee (No. T27-2) and carried out in compliance with the animal care guidelines of Tokushima University and the ARRIVE guidelines **(**PLoS Bio 8(6), e1000412, 2010).

Six-week-old male C57BL/6 mice were subjected to inhalation anesthesia with isoflurane and a stereotactic apparatus was placed in the right brain. With a dental drill, a small hole was bored into the skull 2.0 mm lateral to the bregma. In a malignant glioma model with mouse GSCs, established by Sampetrean and Saya^20^, and Shibao, et al.^21^, GSC progeny cells (1 × 10^3^) in 2 μl of Hank’s balanced salt solution (Sigma-Aldrich) were injected into the right cerebral hemisphere 3 mm below the brain surface, using a 10-μl Hamilton syringe. To examine the anti-tumor effects of SDT, the mice were randomized and treated with 5-ALA, US, or SDT and compared to the non-treated control (Fig. 2). For SDT, a 0.2-ml solution of 5-ALA in PBS was intraperitoneally injected at a dose of 200 mg/kg body weight. Three hours later, the mouse right brain was placed on the stereotactic apparatus and subjected to US imaging (1 MHz, 2 W/cm^**2**^ for 2 min) under inhalation anesthesia. On day 1 after SDT, apoptosis induction by SDT was confirmed and tumor volume on days 3, 7, and 14 and the survival rate were analyzed. Mice were euthanized and their brains were sliced on a brain slicer matrix at 1.0-mm intervals and the tumor volume, represented by the GFP-positive area, was microscopically determined (Keyence BZ-X710, Osaka, Japan).

In addition, to assess the effect of combination therapy of SDT and celecoxib, another set of mice were randomized and treated with vehicle, celecoxib, SDT, or a combination of celecoxib and SDT (Fig. 4). Celecoxib, lysed with dimethyl sulfoxide (DMSO) and hydroxypropyl-β-cyclodextrin (HBC), was injected (i.p.) at a dose of 10 mg/kg consecutively after mouse GSC implantation. Vehicle controls received equivalent doses of DMSO/HBC and normal saline at the same dosing schedule. To validate the efficacy of celecoxib during SDT, tumor volume on day 14 and the survival rate during the observation period were assessed in each group (Fig. 4) as described above.

### Measurement of cellular PpIX levels and SDT

Luminescence was measured 1, 2, 3, 6, 12 or 24 h after treatment of human GBM U251 cells with 1 μM 5-ALA in human GBM U251 cells and GSCs, using the image analyzer in the BZ-X710 microscope (KEYENCE). Thereafter, the viability assessed for 3-48h of GSCs upon combination therapy with SDT and US (1 MHz, 2 W/cm^2^ for 2 min) at 3 h after 5-ALA treatment was assessed after 24 h.

Celecoxib (Sigma-Aldrich, PHR1683) was dissolved in DMSO and supplemented in the culture medium at a final concentration of 60 μM. After 3-h incubation of cells in medium supplemented with 1 μM 5-ALA (SBI ALA promo, Tokyo, Japan), the medium was replaced with fresh complete medium, and the 96-well plate was exposed to LED irradiation (630 nm, 80 mW/cm^2^) for 5 min. The LED light spot was an equally illuminated rectangular spot encompassing the entire culture plate. SDT was performed using the ultrasonic generator UST-770 (ITO Co. Ltd., Tokyo, Japan). In the mouse GSC-bearing glioma model, the tumor area was extracted 3 h after treatment with 200 mg/kg 5-ALA and PpIX levels were analyzed as previously described^20^.

### Quantitative real-time PCR (qRT-PCR)

Total RNA was isolated and extracted using the MagNA Pure RNA isolation kit (Roche, Tokyo, Japan) and the MagNa lyser (Roche), in accordance with the manufacturer’s instructions. We used Transcriptor Universal cDNA Master (Roche) to reverse-transcribe total RNA to cDNA and a LightCycler 2.0 (Roche Diagnostics, Tokyo, Japan) for qRT-PCR. The following primers were used: mouse mdr1, 5′-primer, GGC ATT GCC TAC CTG TTG G-3′; 3′-primer GCT TTC TGT GGA CAC TTC TG, and mouse glyceraldehyde-3-phosphate dehydrogenase (*Gapdh*), 5′-CAG AAC ATC ATC CCT GCA TC-3′ and 5′-CTG CTT CAC CAC CTT CTT GA-3′. The mRNA levels were normalized to those of *Gapdh*. The PCR conditions were as follows: 95 °C for 10 min, followed by 40 cycles at 95 °C for 10 s, 60 °C for 10 s, and 72 °C for 8 s. We subjected 4 samples in each group to the qRT-PCR assay to determine the gene expression levels.

### Western blot analysis

According to our previous study^6^, cells or tissue samples were homogenized in RIPA buffer containing a protease/phosphatase inhibitor cocktail (Cell Signaling Technology, CST, 5872). After 10-min centrifugation at 12,000 rpm, 4 °C, the protein concentration in the supernatants was determined using BCA kit (Thermo Fisher Scientific, USA). Protein (20 or 50 μg) was separated by SDS-PAGE and transferred to polyvinylidene fluoride membranes (immune-blot PVDF membrane, BIO-RAD, Hercules, CA, USA) by electroblotting. Based on the molecular weight marker, each membrane was cut before hybridization. The membranes were immersed in blocking buffer (5% skim milk or 2% BSA in tris-buffered saline, TBS) for 1 h and incubated with primary antibodies: anti-MDR1 (BD Biosciences, NJ, USA, 1:1,000), anti-cCaspase-8, -9, -3 and anti-PARP (Cell Signaling Technology, MA, USA, 1:1,000), anti-AKT2 (Abcam, Cambridge, UK, rabbit, 1:1,000), pAkt (Santa Cruz Biotechnology, CA, USA, rabbit, 1:500), anti-pNF-κB (CST, 1:1000), anti-pI-κB (CST, 1:1000) and β-actin (Sigma-Aldrich, mouse, 1:5000) were diluted in Can Get Signal Solution 1 (Toyobo). After washing in Tween-TBS (T-TBS), the membranes were incubated for 1 h with horseradish peroxidase-conjugated secondary antibodies in Can Get Signal Solution 2 (dilution 1:3000). After washing, the protein-antibody complexes were detected with Amersham ECL prime Western blotting detection reagents (GE Healthcare, UK) using a Lumino image analyzer (Image Quant LAS-4000 mini, GE Healthcare Japan, Tokyo, Japan) and ImageJ 1.52 software (NIH, Bethesda, MD, USA) was used to analyze the protein expression levels*.* Each experiment was repeated four times.

### Immunohistochemistry

Murine tissue samples were fixed with 4% paraformaldehyde and 5-μm-thick frozen sections were mounted on Matsunami adhesive saline-coated glass slides (Matsunami Glass, Tokyo, Japan). As previously reported^6^, human glioma tissue sections from the paraffin-embedded block were dewaxed, rehydrated, and subjected to antigen retrieval. The sections were blocked for 30 min with 1–3% hydrogen peroxide solution, and stained overnight at 4 °C with the following antibodies: anti-MDR1 (D-11) (Santa Cruz Biotechnology, Inc., Dallas, TX, USA, 1:100), anti-COX-2 (Abcam, ab15191), rabbit monoclonal anti-MDR1 (ab170904; 1:100), anti-cleaved caspase-8 (cCasp-8), anti-cCasp-9, anti-cCasp-3, and anti-PARP (CST, 1:1,000). Thereafter, they were incubated with biotinylated secondary antibody (30 min, 30 °C), visualized using DAB buffer tablets, and counterstained with hematoxylin. Photographs were obtained under a light microscope, using KEYENCE BZ-X710.

### Statistical analysis

Survival estimates and median survivals were determined using Kaplan–Meier survival curves. A log-rank (Mantel-Cox) test was performed to determine the *p* values derived from Kaplan–Meier survival curves. To determine statistical significance, between-group comparisons were performed using Student’s *t*-test. For multiple comparisons, one-way ANOVA, followed by the Tukey–Kramer tests. Error bars indicate the standard deviation values. All statistical analyses were performed using JMP 13.2 (SAS Institute Inc.) and the differences with *p* < 0.05 were considered significant.

## Supplementary Information

Supplementary Information.
