# Supplementary material for: Elevated cellular PpIX potentiates sonodynamic therapy in a mouse glioma stem cell-bearing glioma model by downregulating the Akt/NF-κB/MDR1 pathway
Source: Sci Rep. 2021 Jul 23;11:15105. doi: 10.1038/s41598-021-93896-0 (PMC8302615; doi:10.1038/s41598-021-93896-0)
Supplement: Supplementary file 1 — Supplementary Information. [file 41598_2021_93896_MOESM1_ESM.docx]

**Elevated cellular PpIX potentiates sonodynamic therapy** **in a mouse glioma stem cell-bearing glioma model by downregulating the Akt/NF**-**κB/MDR1 pathway**

Kenji Shono^1^, Yoshifumi Mizobuchi^1*^, Izumi Yamaguchi^1^, Kohei Nakajima^1^, Yuri Fujiwara^2^, Toshitaka Fujihara^1^, Keiko Kitazato^1^, Kazuhito Matsuzaki^1^, Yoshihiro Uto^2^, Oltea Sampetrean^3^, Hideyuki Saya^3^, Yasushi Takagi^1^

Supplement
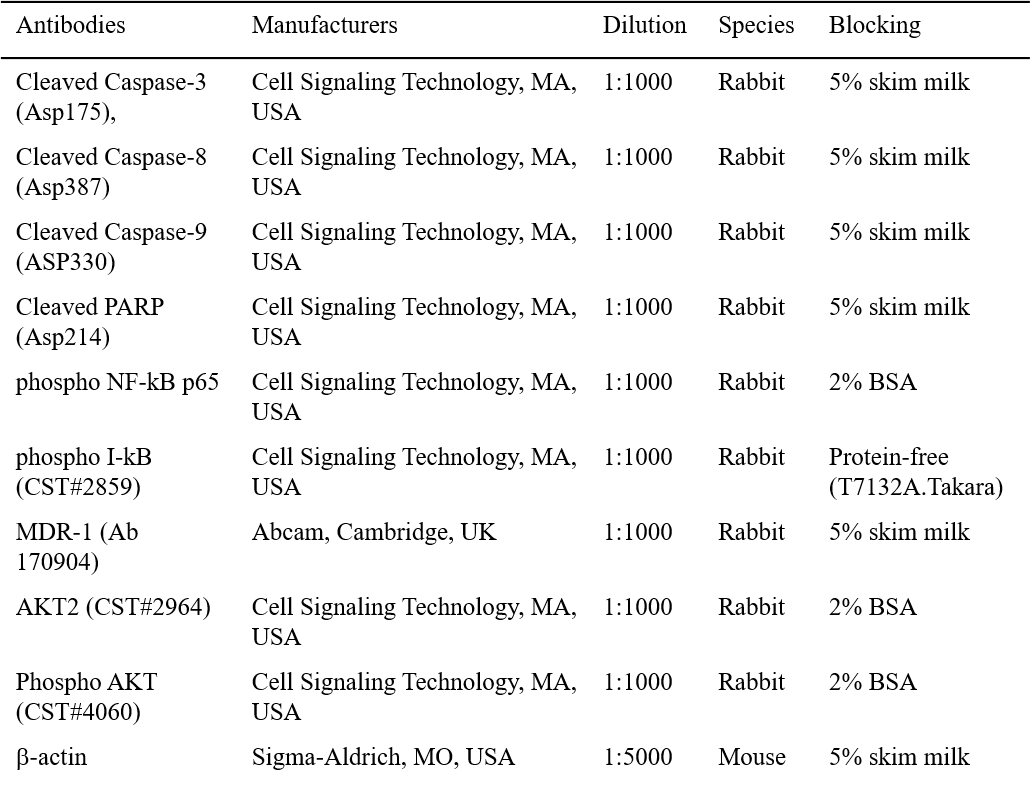
 Table 1　List of antibodies used in western blotting analysis

Supplement Table 2. List of antibodies used in immunohistochemistry


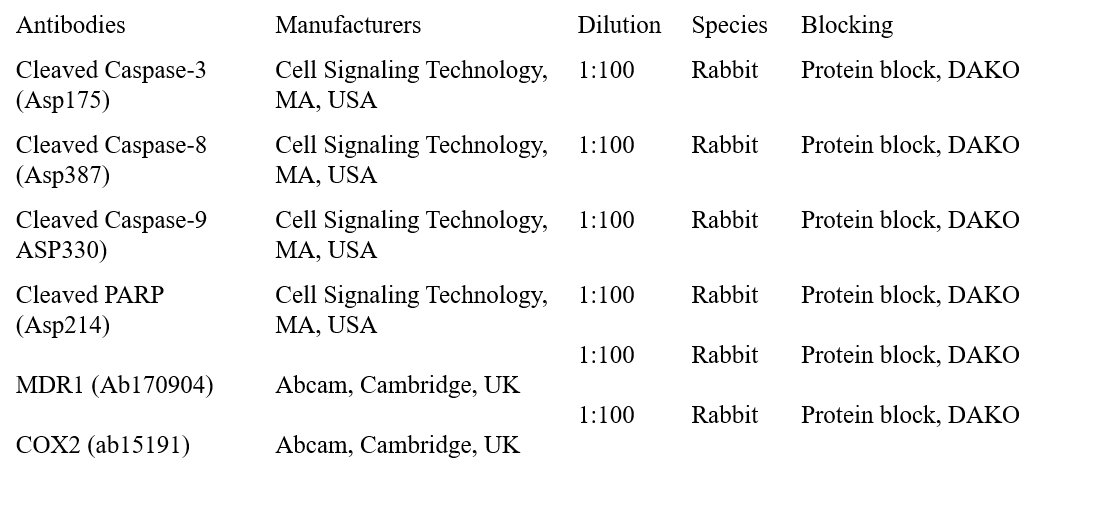


***Supplemental Method***

***Western blot analysis***

As mentioned in method section, each blot membrane was cut horizontally and/or vertically before hybridization with each antibody, based on the molecular weight marker. They were immersed in blocking buffer (5% skim milk, 2% BSA or T-7132A protein-free block (Takara) in tris-buffered saline, TBS) for 1 h and incubated with primary antibodies diluted in Can Get Signal Solution 1 (Toyobo). The protein-antibody complexes were detected with Amersham ECL prime Western blotting detection reagents (GE Healthcare, UK) using a Lumino image analyzer (Image Quant LAS-4000 mini, GE Healthcare Japan, Tokyo, Japan) and ImageJ 1.52 software (NIH, Bethesda, MD, USA) was used to analyze the protein expression levels*.*

**Supplemental figure legends**

Supplement Figure 2.

b. Representative expression of anti-cCaspase-8, -9, -3 and anti-PARP by western blot analysis on day 1 after SDT compared to non-treatment control in a GSC-bearing mouse glioma model. Each arrow indicates molecular weight (Kd).

Supplement Figures 3.

b and c. Changes of MDR1 expression by western blot analysis 24 h after treatment with celecoxib (b) or valspodar, a MDR1 inhibitor (c) compared with DMSO as a vehicle control (VC) in GSCs

Supplement Figure 4.

b. The MDR1 expression by western blot analysis was compared with normal- and tumor brain tissue treated with celecoxib or a vehicle control (VC) included DMSO/HBC.

c. Expression of apoptosis-related molecules at day 1 after SDT compared to VC and the combination with celecoxib and SDT

Supplement Figure 5.

1. Expression of AKT, pAKT, pNF-κB and MDR1 after treatment once a day with celecoxib or vehicle for 7 days in the mouse GSC-bearing glioma model compared to normal mouse brain

b. Protein levels of MDR1 in GSCs treated with or without 1 μM CAPE, a potent and a specific inhibitor of NF-κB activation.

Supplement Figure 2.

b. Representative expression of anti-cCaspase-8, -9, -3 and anti-PARP by western blot analysis on day 1 after SDT compared to non-treatment control in a GSC-bearing mouse glioma model. Each arrow indicates molecular weight (Kd).

Supplement Figures 3.

b and c. Changes of MDR1 expression by western blot analysis 24 h after treatment with celecoxib (b) or valspodar, a MDR1 inhibitor (c) compared with DMSO as a vehicle control (VC) in GSCs

Supplement Figure 4.

b. The MDR1 expression by western blot analysis was compared with normal- and tumor brain tissue treated with celecoxib or a vehicle control (VC) included DMSO/HBC.

c. Expression of apoptosis-related molecules at day 1 after SDT compared to VC and the combination with celecoxib and SDT

Supplement Figure 5.

1. Expression of AKT, pAKT, pNF-κB and MDR1 after treatment once a day with celecoxib or vehicle for 7 days in the mouse GSC-bearing glioma model compared to normal mouse brain.
2. Protein levels of MDR1 in GSCs treated with or without 1 μM CAPE, a potent and a specific inhibitor of NF-κB activation.
